# Transduction as a Potential Dissemination Mechanism of a Clonal *qnrB19*-Carrying Plasmid Isolated From *Salmonella* of Multiple Serotypes and Isolation Sources

**DOI:** 10.3389/fmicb.2019.02503

**Published:** 2019-11-07

**Authors:** Andrea I. Moreno-Switt, David Pezoa, Vanessa Sepúlveda, Iván González, Dácil Rivera, Patricio Retamal, Paola Navarrete, Angélica Reyes-Jara, Magaly Toro

**Affiliations:** ^1^Escuela de Medicina Veterinaria, Facultad de Ciencias de la Vida, Universidad Andres Bello, Santiago, Chile; ^2^Millennium Initiative for Collaborative Research on Bacterial Resistance (MICROB-R), Santiago, Chile; ^3^Facultad de Ciencias, Escuela de Medicina Veterinaria, Universidad Mayor, Santiago, Chile; ^4^Departamento de Medicina Preventiva Animal, Facultad de Ciencias Veterinarias y Pecuarias, Universidad de Chile, Santiago, Chile; ^5^Laboratorio de Microbiología y Probióticos, Instituto de Nutrición y Tecnología de los Alimentos (INTA), Universidad de Chile, Santiago, Chile; ^6^Millennium Nucleus in the Biology of Intestinal Microbiota, Santiago, Chile

**Keywords:** antimicrobial resistance, foodborne diseases, plasmid, quinolones, *qnrB19*, *Salmonella* spp., Chile, plasmid-mediated quinolone resistance

## Abstract

Antimicrobial resistance is an increasing problem worldwide, and *Salmonella* spp. resistance to quinolone was classified by WHO in the high priority list. Recent studies in Europe and in the US reported the presence of small plasmids carrying quinolone resistance in *Enterobacteriaceae* isolated from poultry and poultry products. The aims of this study were to identify and characterize plasmid-mediated quinolone resistance in *Salmonella* spp. and to investigate transduction as a possible mechanism associated to its dissemination. First, we assessed resistance to nalidixic acid and/or ciprofloxacin in 64 *Salmonella* spp. and detected resistance in eight of them. Genomic analyses determined that six isolates of different serotypes and sources carried an identical 2.7-kb plasmid containing the gene *qnrB19* which confers quinolone resistance. The plasmid detected also has high identity with plasmids reported in the US, Europe, and South America. The presence of similar plasmids was later surveyed by PCR in a local *Salmonella* collection (*n* = 113) obtained from diverse sources: food (eggs), wild and domestic animals (pigs, horse, chicken), and human clinical cases. *qnrB19*-carrying plasmids were found in 8/113 *Salmonella* tested strains. A bioinformatics analysis including Chilean and previously described plasmids revealed over 95.0% of nucleotide identity among all the sequences obtained in this study. Furthermore, we found that a *qnrB19*-carrying plasmid can be transferred between *Salmonella* of different serotypes through a P22-mediated transduction. Altogether our results demonstrate that plasmid-mediated quinolone resistance (PMQR) is widespread in *Salmonella enterica* of different serotypes isolated from human clinical samples, wild and domestic animals, and food in Chile and suggest that transduction could be a plausible mechanism for its dissemination. The occurrence of these antimicrobial resistance elements in *Salmonella* in a widespread area is of public health and food safety concern, and it indicates the need for increased surveillance for the presence of these plasmids in *Salmonella* strains and to assess their actual impact in the rise and spread of quinolone resistance.

## Introduction

Antimicrobial resistance is an increasing worldwide problem and a global concern that involves significant health and economic burden (Naylor et al., 2016). Latin America is not an exception: the Pan American Health Organization (PAHO) declared that the spread of pathogens carrying antimicrobial traits challenges both, disease control and disease treatment, and it significantly impacts public health in the region [Pan American Health Organization (PAHO), 2014].

The emergence of antimicrobial resistance in microorganisms occurs naturally; however, the increasing use of antimicrobials promotes the natural selection of resistant bacteria (Holmes et al., 2016). Quinolones are one of the groups of antimicrobials used in humans for the treatment of bacterial infections, and they are also widely used in animal production (Millanao et al., 2011; Singer et al., 2016). Quinolone resistance in enteric pathogens has been described in Latin America [González and Araque, 2013; Pan American Health Organization (PAHO), 2014], and an increase in *Salmonella* spp. resistance to quinolones has been recently reported in Chile [Instituto de Salud Pública de Chile (ISP), 2015] and in the United States (Medalla et al., 2013; Karp et al., 2018).

Antimicrobial resistance to quinolones can be the result of target mutations reducing the drug’s binding to the enzymes gyrase or topoisomerase IV (Hooper and Jacoby, 2016). Additionally, genes harbored in plasmids—such as *qnr* genes—codify for proteins that protect the target enzymes from quinolone action in the phenomena known as plasmid-mediated quinolone resistance (PMQR) (Hooper and Jacoby, 2016). The presence of antimicrobial resistance genes in plasmids is of great concern from a public health perspective because they can easily spread from one bacterium to another through horizontal gene transfer (Rozwandowicz et al., 2018). Three small plasmids carrying the gene *qnrB* have been described since 2010 in South America (Pallecchi et al., 2009; Tran et al., 2012; Cordeiro et al., 2016). The plasmids were obtained from bacteria isolated in Colombia, Peru, and Argentina, and their sizes ranged from 2,699 to 2,750 bp (Karczmarczyk et al., 2010; Pallecchi et al., 2010; Tran et al., 2012). Moreover, some of them can be transferred by conjugation (Andres et al., 2013). Recently, similar plasmids have also been reported in Europe and North America in *Salmonella* isolated from poultry (Fiegen et al., 2017; Tyson et al., 2017).

The aims of this study were to investigate the presence and characteristics of plasmid-mediated quinolone resistance in *Salmonella* spp. of different serotypes and sources, and to investigate whether transduction could be a potential mechanism associated to its dissemination.

## Materials and Methods

### Strains

*Salmonella enterica* (*S. enterica*; *n* = 64) from different serotypes were isolated from wild birds (*n* = 28), human clinical cases (*n* = 23), eggs (*n* = 9), and sea lions (*n* = 4). Isolates were collected between 2009 and 2012 from different locations along Chile. Isolate’s information, including genomic sequences, was reported in previous publications (Toro et al., 2015, 2016) (Supplementary Table S1). Additionally, 113 *S. enterica* strains from our historic collection were recovered to survey for the presence of *qnrB19*-carrying plasmids through PCR as described below. These *S. enterica* belong to different serotypes and were originally isolated from pigs (*n* = 9), horses and their environment (*n* = 25), cattle (*n* = 3), poultry and poultry farm environments (*n* = 29), wild birds (*n* = 18), wild reptiles (*n* = 17), and Chilean mouse opossum (*n* = 12) (Supplementary Table S2). Strains were recovered in trypticase soy agar (TSA) agar (BD, Franklin Lakes, NJ) and after an overnight incubation, DNA was purified with the Qiagen DNeasy Blood and Tissue kit (Qiagen, Valencia, CA).

### *In vitro* Determination of Quinolone Resistance

*Salmonella* isolates (*n* = 64) were tested for antimicrobial susceptibility against the quinolones nalidixic acid (NAL) and ciprofloxacin (CIP), and to other 12 drugs included in the NARMS program at the US FDA Center for Veterinary Medicine (CVM) following their standard protocols (Karp et al., 2017). In brief, minimum inhibitory concentrations (MICs) were determined by the microdilution method through the Sensititre automated microbial susceptibility system (Thermo Fisher Scientific, Waltham, MA) at the Laboratory of the Center for Veterinary Medicine, U.S. Food and Drug Administration. *Escherichia coli* ATCC 25922, *Enterococcus faecalis* ATCC 29212, *Staphylococcus aureus* ATCC 29213, and *Pseudomonas aeruginosa* ATCC 27853 were used as controls for antimicrobial MIC determinations. The results were interpreted according to the Clinical and Laboratory Standard Institute MIC standards [Clinical Laboratory Standards Institute (CLSI), 2015].

### *In silico* Determination of Antimicrobial Resistance

Genomic data for *Salmonella* (*n* = 64) isolates was previously reported (Toro et al., 2015, 2016). In this study, we investigated the presence of acquired antimicrobial resistance genes and known chromosomal point mutations linked to antimicrobial resistance in quinolone-resistant strains using the ResFinder 3.0 server [Center for Genomic Epidemiology (CGE), Technical University of Denmark, Lyngby, Denmark, https://cge.cbs.dtu.dk/services/ResFinder/] (Zankari et al., 2012). Gene identification threshold was set at 60% identity and 60% minimum length.

### Plasmid Sequence Study

Nucleotide sequences of contigs containing quinolone antimicrobial resistance genes—as identified with ResFinder (Supplementary Table S5)—were used to search for identities at the NCBI database with the BLAST algorithm using the highly similar sequences setting (megablast). Previously reported plasmids with high identity values (*n* = 6) were found and used for genetic comparison (Supplementary Table S3). The plasmid set was compared by aligning their nucleotide sequences in Geneious Prime 2019.1.1 (Biomatters, New Zealand) with the ClustalW (Larkin et al., 2007) plug-in with default parameters. A phylogenetic reconstruction was crafted from the alignment with the RAxML plug-in using the “Rapid bootstrapping and search for the best scoring Maximum-likelihood tree” option with 1,000 replicates (Stamatakis et al., 2008), and a consensus tree was obtained.

### Screening Study for Small Plasmids Carrying the *qnrB19* Gene

**Figure 1:**
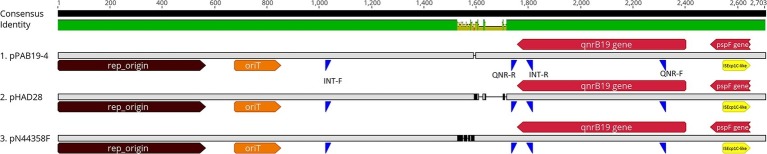
Linear representation of plasmids carrying the *qnrB19* gene. Plasmids pPAB19-4, pHAD28, and pN44358F were selected to represent different clusters resulting from the phylogenetic analysis (Supplementary Figure S1). Alignment was crafted with the ClustalW plug-in in Geneious Prime 2019 using default parameters. Consensus identity bar: green represents 100% identity among all sequences, green-brown indicates 30 to <100% identity, and red shows <30% identity. Genomic features are represented by different colors; brown: plasmid origin of replication (rep_origin; nt 1-564); orange: Origen of transfer (oriT; nt 677-851); red: genes *qnrB*-19 (nt 2,402-1,758) and gene *psp*F (nt2,647-2,496); yellow: ISEcp1C-like insertion sequence (nt 2,512-2,409). Primer alignment sites are represented in blue: INT-F and INT-R represent primers for the intergenic region. QNR-F and QNR-R represent primers for the *qnrB*-19 gene region.

We determined the presence of plasmids similar to pPAB19-4 in 113 different *Salmonella* isolates from our historic collection through PCR. For primer design, three similar but distinct plasmids were aligned (i.e., pPAB19-4, pHAD28, and pN44358F; Supplementary Figure S1, Supplementary Table S3) with ClustalW in Geneious using default parameters, and potential target regions were identified (Figure 1). Finally, two sets of primers were designed: the first set of primers amplified a 589-bp region from the 5′ end of the *qnrB19* gene to the immediate intergenic region (QNR-F: 5′ ACTGCGATTTTTCAGGTGCC 3′; QNR-R: 5’CATCTCCCGGTGTAAACGCT 3′), and the second set of primers amplified a variable-size plasmid backbone region (704–790 bp) from the distant intergenic region to the 3’end of the *qnrB19* gene (Int-F 5′ CTGACAAACTTGACGCCTGC 3′; Int-R5′ GACAGCTACCAGGCATCGTT 3′) (Figure 1). GoTaq DNA Polymerase (Promega, San Luis Obispo, CA) was used for each PCR, and the following conditions were used: initial denaturation at 95°C for 5 min, 30 cycles of denaturation at 95°C for 30 s, annealing at 57°C for 30 s, extension of 72°C for 1 min, and a final extension of 72°C for 7 min. PCRs were amplified using an Axygen MaxyGene II Thermal Cycler (Corning, Corning, NY).

### Genomic Comparison of Small Plasmids Carrying the *qnrB19* Gene

Newly identified *qnrB*-19-carrying isolates (*n* = 4/113) were submitted for whole genome sequencing (WGS). WGS of two of these isolates (DR-021 with number CFSAN035154 and DR-022 with number CFSAN035155) was described in a previous publication (Toro et al., 2018). The other two isolates (i.e., DR-039 and DR-040; acc. Numbers: SAMN11569606 and SAMN11569607, respectively) were sequenced at MicrobesNG at the School of Biosciences, University of Birmingham (Birmingham, United Kingdom) (Supplementary Table S2). Libraries were created with the nextera-XT library prep kit, and sequencing was performed in an Illumina HiSeq plaform using 250-bp paired end protocol to a minimum coverage of 30x. Genomes were *de novo* assembled from raw reads with SPAdes version 3.1.0 (Bankevich et al., 2012), and annotation was done at the RAST annotation server (Aziz et al., 2008). Plasmid sequences were obtained from assembled genomes using the “map to reference” utility in Geneious Prime, and pPAB19-4 sequence (acc. Nbr: JN995611.1) was selected as a reference. Later, a comprehensive comparative analysis including all *qnrB19*-carrying plasmids was run in Geneious Prime 2019.1.1 (Biomatters, New Zealand) with the ClustalW (Larkin et al., 2007) and with MAUVE (Darling et al., 2004) plug-ins using default parameters. Additionally, plasmid sequences were screened for phage DNA using PHASTER 2.7 (Arndt et al., 2016), and we also searched for type IV secretion system (T4SS) elements for all 12 genomes containing pPAB-19-like Chilean plasmids using ConjScan in the Pasteur Galaxy platform v1.0.2 (Abby et al., 2016).

### Plasmid Transduction Assays

Transduction experiments were performed according to the protocol previously described by Maloy et al. (1996). *Salmonella* Heidelberg SAL4674 (CFSAN024772)—a *qnrB19*-plasmid carrier strain sequenced in this study—was used as donor, and phage P22HT (high transduction frequency) was used as transducing phage. Briefly, the donor was grown overnight in Luria Bertani broth (LB) broth (Difco; Detroit, MI) supplemented with 15 μg/ml of NAL (Sigma Chemical Co, Saint Louis, MO). Then, 200 μl of the *Salmonella* culture was mixed with 1 ml of P22 broth (LB broth containing 0.2 mg/ml MgSO_4_ × 7H_2_O, 2 mg/ml citric acid, 13.1 mg/ml K_2_HPO_4_ × 3H_2_O, 3.5 mg/ml NaNH_4_HPO_4_ × 4H_2_O, 2 mg/ml glucose, and 100 μl of P22 phage lysate), and the mixture was incubated overnight at 37°C with agitation. To obtain the phage lysate, the culture was centrifuged at 13,000 rpm for 2 min, and 100 μl of chloroform was added to the supernatant. Phage lysates were kept at 4°C until its use. For transduction experiments, *Salmonella* Typhimurium 14,028 s marked with a chloramphenicol resistance cassette in the *phoN* gene (recipient strain) was grown overnight at 37°C with agitation, and 200 μl of this culture was mixed with 20 μl of the P22 phage lysate obtained from the donor strain. Then, the mixture was incubated at room temperature for 15 min to allow phage adsorption, and 1 ml of LB broth was added. The mixture was incubated at 37°C for 60 min to allow phenotypic expression of the antibiotic marker. Later, the mixture was centrifuged at 13,000 rpm for 2 min, and the supernatant was discarded. The cell pellet was suspended in 1 ml of LB broth, and serial 10-fold dilutions were spread on LB agar plates containing NAL (15 μg/ml) and chloramphenicol (20 μg/ml) for determination of transductants. Negative controls, i.e., experiments run without the phage or bacteria, were included as described by Maloy et al. (1996). The transduction frequency was calculated as the ratio of the number of transductants (CFU) obtained in the receptor strain to the number of plaque-forming units (PFUs) in the transduction mixture of three independent experiments. The plasmid transduction to the recipient strain was verified by PCR amplification with the same two sets of primers mentioned in the previous section.

## Results

### *In vitro* Determination of Antimicrobial Resistance

The antimicrobial susceptibility was evaluated for 64 *Salmonella* strains. *In vitro* tests showed that eight *Salmonella* spp. had reduced susceptibility to quinolones: of those, two strains (SAL4629 and SAL4630) were resistant to CIP (MIC ≥ 1.0 μg/ml), and five (SAL 4629, SAL4630, SAL4675, SAL4676, and SAL4679) were resistant to NAL (MIC ≥32 μg/ml) (Table 1). In addition, strain SAL4630, isolated from eggs, was also resistant to tetracycline, trimethoprim-sulfamethoxazole and sulfidoxazole. Antimicrobial susceptibility results for strains SAL4629 and SAL4630 were reported in Toro et al., 2016.

**Table 1:** Minimum inhibitory concentration (MIC) to quinolones of *Salmonella* strains isolated in Chile.

Strain	Acc. number	Serotype	Sequence type	Source	MIC CIP (μg/ml)	MIC NAL (μg/ml)	Antimicrobial resistance genes
SAL4629^¥^	LILV00000000	Enteritidis	11	Poultry	1.0	32	qnrB19^*^
SAL4630^¥^	LILU00000000	Enteritidis	11	Poultry	1.0	32	qnrB19^*^
SAL4674	JWQH00000000	Heidelberg	15	Kelp Gull	0.5	16	qnrB19^*^
SAL4675	JWQG00000000	Heidelberg	15	Kelp Gull	0.25	> 32	*gyrA* aa 83 F/S^**^
SAL4676	JWQF00000000	Heidelberg	15	Kelp Gull	0.25	> 32	*gyrA* aa 83 F/S^**^
SAL4678	JWQE00000000	Heidelberg	15	Human	0.5	16	qnrB19^*^
SAL4679	JWQD00000000	Heidelberg	15	Human	0.5	32	qnrB19^*^
SAL4688	JWRD00000000	Senftenberg	14	Kelp Gull	0.5	16	qnrB19^*^

*CIP, ciprofloxacin; NAL, nalidixic acid*.

*Antimicrobial susceptibility ranges. CIP: susceptible (≤ 0.06); non-susceptible (0.06–1); resistant (≥ 1)*.

*NAL: susceptible (≤ 16); resistant (≥ 32)*.

* *Acquired resistance genes searched using ResFinder tool, DTU. *qnrB19*: acquired antimicrobial resistance gene, plasmid borne*.

** *Genomic *gyrA* gene: mutation in aminoacid 83*.

¥ *Data published in Toro et al., 2016*.

### *In silico* Determination of Quinolone Resistance

We identified genes or known gene mutations for antimicrobial resistance in all eight strains displaying quinolone reduced susceptibility or resistance (Table 1). The mutation *gyrA* S83F, previously linked to quinolone resistance (Piddock et al., 1998; Reche et al., 2002; Tyson et al., 2015), was found in two of the eight later isolates (Table 1). Interestingly, all six strains that lacked the *gyr*A mutation but were resistant or had reduced susceptibility to quinolones carried the gene *qnrB19*, which has been linked to reduced susceptibility to quinolones in Gram negative bacteria (Tran et al., 2012). *In silico* sequence typing and serotyping analysis showed that these six strains belonged to different sequence type (ST) and to different serotypes (Table 1).

### Plasmid Sequence Study

Our analysis revealed that the *qnrB19* gene was harbored in a 2,702-bp plasmid 100% identical among all six sequenced strains (Table 1; Supplementary Figure S1). A search within the NCBI database also identified that the sequence was 100% identical to plasmid pPAB19-4 (accession number: JN995611), which was first described by Tran et al. (2012) in a clinical *Salmonella* sp. isolate obtained in Argentina. Several other plasmids from the database showed high identity values, and six were selected for phylogenetic analysis (Supplementary Figures S1, S2, Supplementary Table S3). The analysis revealed that 12 plasmid sequences (six from our study and six from the NCBI database) grouped in three clusters: one cluster included plasmid pPAB19-4, all six Chilean plasmids, and plasmid pMK101 reported in Colombia (Karczmarczyk et al., 2010). Plasmids pECY6-7 (Peru), pSGI5 (The Netherlands), and pN44358F (US) clustered together (Hammerl et al., 2010; Pallecchi et al., 2010; Tyson et al., 2017). Finally, plasmid pHAD28 reported recently in Germany did not cluster with other plasmids (Fiegen et al., 2017) (Supplementary Figure S1). Differences among these three clusters were located in an intergenic region upstream of the *qnrB* gene (Supplementary Figures S1, S2). Finally, plasmids pPAB19-4, pN44358F, and pHAD28 were selected as representative of each cluster and used for primer design (Figure 1).

### Plasmid Screening by PCR in a Historic *Salmonella* Collection

PCR amplification with the two sets of primers (Figure 1) identified the presence of the *qnrB19*-carrying plasmids in 8/113 *Salmonella* strains isolated from chicken (1/29), pigs (3/9), horse (1/25), wild birds (2/18), and one from a chicken farm environment (1/1). *qnrB*-19-carrying plasmids were detected in eight *Salmonella* isolates of serotypes Hadar, Typhimurium, and serogroup B *Salmonella*, and were obtained in Chile from 2012 to 2017 (Supplementary Table S2).

### Genomic Comparison of Small Plasmids Carrying the *qnrB19* Gene

**Figure 2:**
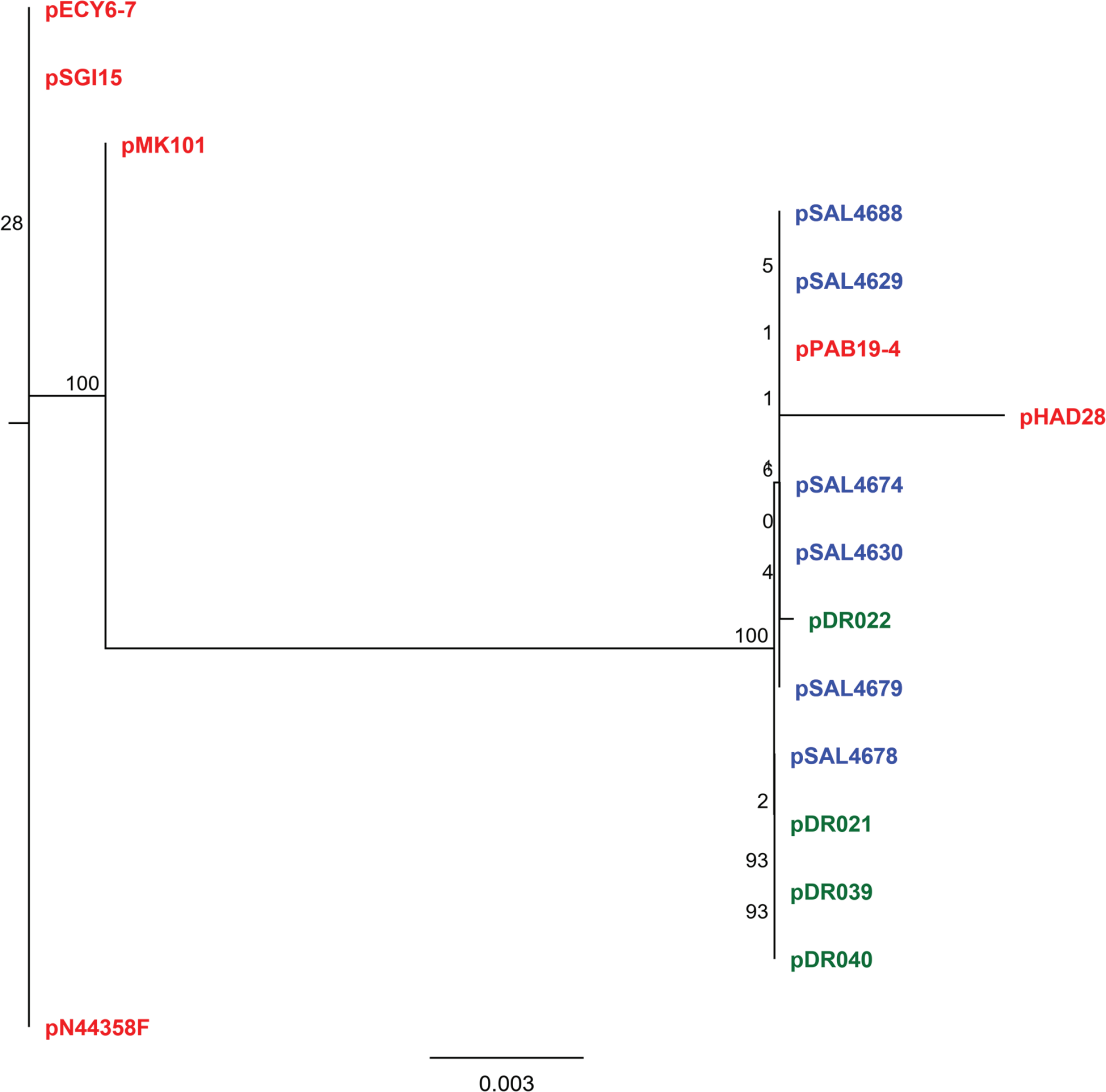
Phylogenetic tree of 16 pPAB-19-like plasmids inferred by maximum-likelihood analysis The analysis included 16 plasmids including six previously described plasmids and 10 plasmids described in the present study. Phylogeny was inferred with RAxML in Geneious Prime (Biomatters, New Zealand) with 1,000 replicates. Colored names represent plasmid sequence source. Red: data from NCBI; blue: genomes reported in Toro et al. (2015) and Toro et al. (2016); green: this study.

**Figure 3:**
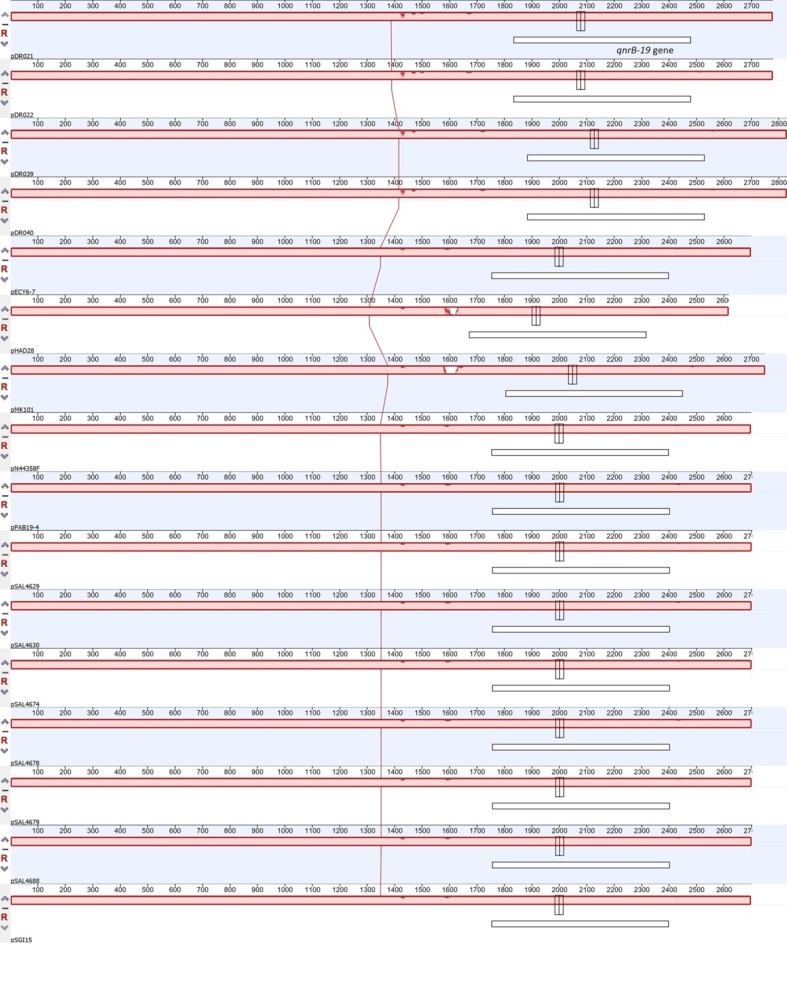
Whole plasmid sequence comparison of 16 *qnrB*-19-like plasmids. The analysis included 16 plasmids including six previously described plasmids and 10 plasmids described in the present study. Whole plasmid sequence comparison was performed with the Mauve plug-in in Geneious Prime with default settings. White box represents location of the *qnrB*-19 gene in each plasmid sequence. Red filled bars represent identity among tested sequences.

We sequenced four of the isolates containing *qnrB*-19 plasmids from our historic collection and added their plasmid sequences (pDR-021, pDR-022, pDR-039, and pDR-040) to the general analysis. The complete set of 16 plasmid sequences (six from the NCBI database, six from *Salmonella* genomes previously reported in Toro et al., 2015, and Toro et al., 2016, and four plasmid sequences from our historic collection) was compared showing that nucleotide differences were of a few nucleotides and hosted in the same intergenic region (Figures 2, 3; Supplementary Table S4). All Chilean plasmids clustered together with plasmid pPAB-19. Newly identified plasmid sequences had over 95% identity to pPAB19-4: plasmids pDR-039 and pDR-040 had 95.5% identity to pPAB19-4, and plasmids pDR-021 and pDR-022 had 97.2% identity (Figure 2; Supplementary Table S4). Differences detected among plasmid sequences were 50-bp or 127-bp insertions in the intergenic region starting at nucleotide 1,486 of pPAB19-4 (Figure 3; Supplementary Figure S3). Additional characterization showed that none of plasmids was identified as similar to phage or prophage sequences at BLAST, and the PHASTER screening showed absence of phage DNA in these sequences. No T4SS elements were found either in the *Salmonella* genomes carrying pPAB19-4-like plasmids. Presence of other plasmid replicons varied (Supplementary Table S4).

### Phage-Mediated Dissemination of the pPAB19-4 Plasmid

It has been reported that P22- and P22-like bacteriophages are capable of efficiently transferring both chromosomal fragments and low-copy number plasmids in *Salmonella* (Mann and Slauch, 1997; Schmieger and Schicklmaier, 1999). Therefore, to investigate a potential mechanism by which pPAB19-4 disseminates through different *Salmonella* serotypes, P22 transduction assays were performed. The transduction frequency was determined as 1.63 × 10^−7^ cfu/pfu (SD ± 6.5 × 10^−6^) and none of the negative controls showed transductants. These results showed that P22 bacteriophage efficiently transfers the plasmid from *S.* Heidelberg donor strain to the recipient *S.* Typhimurium strain.

## Discussion

Antimicrobial resistance is one of the main concerns in public health. WHO defined this problem as high priority and promotes improving research and surveillance as measures to attack the problem (Tacconelli et al., 2017). Quinolones are a group of antimicrobials that are used to treat human and animal bacterial infections; therefore, resistance to these drugs is of great concern (Tacconelli et al., 2017). In Chile, recent studies have determined that quinolone resistance is on the rise [Instituto de Salud Pública de Chile (ISP), 2015], and a recent study reported that *Salmonella* susceptibility to quinolones is decreasing in the US (Karp et al., 2018). In the present study, we found that a small plasmid harboring the gene *qnrB19* was the main responsible for quinolone antimicrobial resistance in six *Salmonella* spp. isolates. Strains carrying the plasmid showed reduced susceptibility or resistance to CIP, and three of them were also resistant to NAL (Table 1). Traditionally, plasmid-mediated quinolone resistance (PMQR) has been linked to low-level quinolone resistance, promoting the selection of quinolone-resistant strains (Rodríguez-Martínez et al., 2016). Also, recent studies have described the presence of plasmids carrying the *qnrB19* gene as responsible for quinolone resistance in *Salmonella* spp. isolated in Europe and the US from poultry and swine sources (Fiegen et al., 2017; Tyson et al., 2017). Similar plasmids have been found in *E. coli* from human and food animals from different geographic regions as well (Hammerl et al., 2010; Jones-Dias et al., 2013; Cummings et al., 2017).

Genes of the *qnr* family can be hosted in small and large plasmids (Hammerl et al., 2010; Tran et al., 2012). The gene *qnrB19* was first described in a *E. coli* isolated from pigs in Guandong, China (Yue et al., 2008), and different plasmids carrying the same gene were reported in Latin-American countries such as Peru, Bolivia, and Colombia (Pallecchi et al., 2010; Tran et al., 2012). Furthermore, an identical plasmid (pPAB19-4) to the one we found in this work was first isolated in Argentina (Tran et al., 2012) from a *Salmonella* sp. clinical isolate, and a very similar plasmid (pSGI15; FN428572) was also found in *Salmonella* Typhimurium from a human clinical case in the Netherlands (Hammerl et al., 2010). Moreover, another identical plasmid was recently isolated from pork products and swine in the US (Tyson et al., 2017). We found also two other highly similar *qnrB*-19 gene-carrying plasmids; identity to pPAB19-4 was tested through different software, and we detected that when differences existed, they were concentrated in the intergenic region and were indels of 77–137 bp, which were not coding regions, and all plasmid features were the same with pPAB19-4 (Supplementary Figure S3).

Interestingly, strains isolated in this study and carrying pPAB19-4-like plasmids were isolated from *Salmonella* of diverse sources and different serotypes (Table 1; Supplementary Tables S1, S2): *S.* Enteritidis strains were isolated from eggs (chicken), *S.* Heidelberg and *S.* Senftenberg strains were isolated from Kelp gull, and *S.* Heidelberg strain was isolated from human clinical cases. *S.* Typhimurium from pigs and *S.* Hadar from chicken also contained similar plasmids. This suggests that this plasmid is probably being transmitted among diverse *Salmonella* lineages by HGT. Supporting this hypothesis, isolates of *Salmonella* Heidelberg and Senftenberg carrying this plasmid have been isolated from swine and pork products in the United States (Tyson et al., 2017).

The widespread presence of pPAB19-4-like plasmids among diverse *Salmonella* serotypes, hosts, years, and geographic locations poses a risk for global human and animal populations. A better understanding of the mechanism involved in the spread of these plasmids could be used to understand their dissemination in the environment. Since unrelated *Salmonella* serotypes and *E. coli* have carried identical plasmids, it was plausible to think that horizontal gene transfer mechanisms were involved on their dissemination. The pPAB19-4 plasmid is small (2.7 kb) and lacks *mob* and *tra* genes, therefore, self-conjugation is not possible (Tran et al., 2012); for this reason, we did not include DNAse treatment in our experiments. A similar plasmid (pPAB19-2) was transferred by conjugation (Andres et al., 2013), suggesting that more than one mechanism of horizontal gene transfer is possible in these types of plasmids. Our results demonstrated that pPAB19-4 plasmids can be transferred from S. Heidelberg to *S*. Typhimurium by transduction assisted by a P22 bacteriophage. Transduction frequency reported in the current study (1 transducent in 10^6^ phage) is similar to that reported in previous studies (Mašlanová et al., 2016; Varga et al., 2016). Importantly, our study shows transduction in experimental conditions, indicating that transduction is another plausible mechanism for pPAB19-4-like plasmids spread in the environment.

Our data demonstrates that the pPAB19-4 plasmid confers antimicrobial resistance to *Salmonella* able to cause human disease (Table 1); therefore, surveillance of the genetic trait in different hosts and environments—including wild and domestic animals, foods and the environment—will be fundamental to understand its relevance in the spread of quinolone resistance. This will be of special importance in regions of the world where the plasmid has not been described yet. Additionally, we believe that it will be important to assess the role of migratory birds and wild fauna in spreading these elements and the associated antimicrobial resistance phenotypes since we found the plasmid in *Salmonella* isolated from gulls. Likewise, we detected the plasmid in *Salmonella* isolated from 2009 to 2015 in Chile, and it was also identified in the US in *Salmonella* isolated in 2013 and 2014 (Tyson et al., 2017). Taken together, evidence suggests that this plasmid has been circulating for at least 8 years in several countries, and that it is spreading to other parts of the world.

In conclusion, the pPAB19-4-like plasmids could play an important role in antimicrobial resistance worldwide. Since the plasmid can be transferred between different salmonellae circulating in the food chain, wild animals, and human clinical cases, it is important to start its active surveillance and to explore the presence of diverse mobile genetic elements carrying this and other resistance genes impacting public health.

## Data Availability Statement

The datasets generated for this study can be found in the NCBI accession numbers: SAMN11569606, SAMN11569607.

## Bioethics Statements

*Salmonella enterica* (*S. enterica*; *n* = 64) isolates from wild birds (*n* = 28), human clinical cases (*n* = 23), eggs (*n* = 9), and sea lions (*n* = 4) were collected between 2009 and 2012 from different locations along Chile. Isolate’s information, including genomic sequences, was reported in previous publications (Toro et al., 2015, 2016) (Supplementary Table S1). The historic strain collection used in this study was obtained in a previous study by AM-S, approved by the University Andrés Bello Bioethics Committee, Santiago, Chile.

## Author Contributions

AM-S wrote the manuscript, analyzed data, and designed the study. DP, VS, and IG performed plasmid transduction assays. DR performed laboratory experiments. PR provided material and wrote the manuscript. PN and AR-J critically reviewed the manuscript. MT wrote the manuscript, performed data analysis, and conceived the study.

### Conflict of Interest

The authors declare that the research was conducted in the absence of any commercial or financial relationships that could be construed as a potential conflict of interest.
