# Corrigendum: Transduction as a Potential Dissemination Mechanism of a Clonal *qnrB19*-Carrying Plasmid Isolated From *Salmonella* of Multiple Serotypes and Isolation Sources

**DOI:** 10.3389/fmicb.2020.00547

**Published:** 2020-04-07

**Authors:** Andrea I. Moreno-Switt, David Pezoa, Vanessa Sepúlveda, Iván González, Dácil Rivera, Patricio Retamal, Paola Navarrete, Angélica Reyes-Jara, Magaly Toro

**Affiliations:** ^1^Escuela de Medicina Veterinaria, Facultad de Ciencias de la Vida, Universidad Andres Bello, Santiago, Chile; ^2^Millennium Initiative for Collaborative Research on Bacterial Resistance (MICROB-R), Santiago, Chile; ^3^Facultad de Ciencias, Escuela de Medicina Veterinaria, Universidad Mayor, Santiago, Chile; ^4^Departamento de Medicina Preventiva Animal, Facultad de Ciencias Veterinarias y Pecuarias, Universidad de Chile, Santiago, Chile; ^5^Laboratorio de Microbiología y Probióticos, Instituto de Nutrición y Tecnología de los Alimentos (INTA), Universidad de Chile, Santiago, Chile; ^6^Millennium Nucleus in the Biology of Intestinal Microbiota, Santiago, Chile

**Keywords:** antimicrobial resistance, foodborne diseases, plasmid, quinolones, *qnrB19*, *Salmonella* spp., Chile, plasmid-mediated quinolone resistance

In the original article, “Andres et al. (2013)” was not cited and referenced in the article. The citation has now been inserted in the **Introduction**, paragraph three and in the **Discussion** section, paragraph four and should read:

“Antimicrobial resistance to quinolones can be the result of target mutations reducing the drug's binding to the enzymes gyrase or topoisomerase IV (Hooper and Jacoby, 2016). Additionally, genes harbored in plasmids—such as *qnr* genes—codify for proteins that protect the target enzymes from quinolone action in the phenomena known as plasmid-mediated quinolone resistance (PMQR) (Hooper and Jacoby, 2016). The presence of antimicrobial resistance genes in plasmids is of great concern from a public health perspective because they can easily spread from one bacterium to another through horizontal gene transfer (Rozwandowicz et al., 2018). Three small plasmids carrying the gene *qnrB* have been described since 2010 in South America (Pallecchi et al., 2009; Tran et al., 2012; Cordeiro et al., 2016). The plasmids were obtained from bacteria isolated in Colombia, Peru, and Argentina, and their sizes ranged from 2,699 to 2,750 bp (Karczmarczyk et al., 2010; Pallecchi et al., 2010; Tran et al., 2012). Moreover, some of them can be transferred by conjugation (Andres et al., 2013). Recently, similar plasmids have also been reported in Europe and North America in *Salmonella* isolated from poultry (Fiegen et al., 2017; Tyson et al., 2017).”

and

“The widespread presence of pPAB19-4-like plasmids among diverse *Salmonella* serotypes, hosts, years, and geographic locations poses a risk for global human and animal populations. A better understanding of the mechanism involved in the spread of these plasmids could be used to understand their dissemination in the environment. Since unrelated *Salmonella* serotypes and *E. coli* have carried identical plasmids, it was plausible to think that horizontal gene transfer mechanisms were involved on their dissemination. The pPAB19-4 plasmid is small (2.7 kb) and lacks *mob* and *tra* genes, therefore, self-conjugation is not possible (Tran et al., 2012); for this reason, we did not include DNAse treatment in our experiments. A similar plasmid (pPAB19-2) was transferred by conjugation (Andres et al., 2013), suggesting that more than one mechanism of horizontal gene transfer is possible in these types of plasmids. Our results demonstrated that pPAB19-4 plasmids can be transferred from *S*. Heidelberg to *S*. Typhimurium by transduction assisted by a P22 bacteriophage. Transduction frequency reported in the current study (1 transducent in 10^6^ phage) is similar to that reported in previous studies (Mašlanová et al., 2016; Varga et al., 2016). Importantly, our study shows transduction in experimental conditions, indicating that transduction is another plausible mechanism for pPAB19-4-like plasmids spread in the environment.”

The authors apologize for this error and state that this does not change the scientific conclusions of the article in any way. The original article has been updated.
