# Supplementary material for: Transduction as a Potential Dissemination Mechanism of a Clonal qnrB19-Carrying Plasmid Isolated From Salmonella of Multiple Serotypes and Isolation Sources
Source: Front Microbiol. 2019 Nov 7;10:2503. doi: 10.3389/fmicb.2019.02503 (PMC6854032; doi:10.3389/fmicb.2019.02503)
Supplement: Supplementary file 1 [file Data_Sheet_1.docx]

**Supplementary Material.**

Moreno Switt et al., 2019

**Supplementary Table 1.** Information for strains in the study for antimicrobial resistance test and whole genome sequencing (64).

| **Strain name** | **CFSAN number** | **Accession number** | **Original Name** | **Source** | **Serotype** | **Isolation year** |
| --- | --- | --- | --- | --- | --- | --- |
| SAL4628 | CFSAN024726 | LIIZ00000000 | SEN2 | Eggs | Enteritidis | 2009 |
| SAL4629 | CFSAN024727 | LILV00000000 | SEN5 | Eggs | Enteritidis | 2009 |
| SAL4630 | CFSAN024728 | LILU00000000 | SEN7 | Eggs | Enteritidis | 2009 |
| SAL4631 | CFSAN024729 | LILT00000000 | SEN21 | Human | Enteritidis | 2010 |
| SAL4632 | CFSAN024730 | LILS00000000 | SEN22 | Human | Enteritidis | 2010 |
| SAL4633 | CFSAN024731 | LILR00000000 | SEN31 | Eggs | Enteritidis | 2011 |
| SAL4634 | CFSAN024732 | LILQ00000000 | SEN47 | Eggs | Enteritidis | 2012 |
| SAL4635 | CFSAN024733 | LILP00000000 | SEN49 | Human | Enteritidis | 2012 |
| SAL4636 | CFSAN024734 | LILO00000000 | SEN53 | Human | Enteritidis | 2012 |
| SAL4637 | CFSAN024735 | LILN00000000 | SEN63 | Human | Enteritidis | 2012 |
| SAL4638 | CFSAN024736 | LIMD00000000 | SEN71 | Human | Enteritidis | 2012 |
| SAL4639 | CFSAN024737 | LIMC00000000 | SEN72 | Human | Enteritidis | 2012 |
| SAL4640 | CFSAN024738 | LIMB00000000 | SEN73 | Human | Enteritidis | 2012 |
| SAL4641 | CFSAN024739 | LIMA00000000 | SEN77 | Human | Enteritidis | 2012 |
| SAL4642 | CFSAN024740 | LILZ00000000 | SEN78 | Human | Enteritidis | 2012 |
| SAL4643 | CFSAN024741 | LILY00000000 | SEN79 | Human | Enteritidis | 2012 |
| SAL4644 | CFSAN024742 | LILX00000000 | SEN82 | Eggs | Enteritidis | 2012 |
| SAL4645 | CFSAN024743 | LILW00000000 | SEN87 | Eggs | Enteritidis | 2012 |
| SAL4646 | CFSAN024744 | LIOP00000000 | SEN88 | Eggs | Enteritidis | 2012 |
| SAL4647 | CFSAN024745 | LIOQ00000000 | SEN89 | Eggs | Enteritidis | 2012 |
| SAL4648 | CFSAN024746 | LIOR00000000 | SEN95 | Franklin gull | Enteritidis | 2011 |
| SAL4649 | CFSAN024747 | LIOS00000000 | SEN97 | Kelp gull | Enteritidis | 2012 |
| SAL4650 | CFSAN024748 | LIOZ00000000 | SEN98 | Kelp gull | Enteritidis | 2012 |
| SAL4651 | CFSAN024749 | LIOY00000000 | SEN99 | Kelp gull | Enteritidis | 2012 |
| SAL4652 | CFSAN024750 | LIPA00000000 | SEN101 | Kelp gull | Enteritidis | 2012 |
| SAL4653 | CFSAN024751 | LIOX00000000 | SEN107 | Kelp gull | Enteritidis | 2012 |
| SAL4654 | CFSAN024752 | LIOW00000000 | SEN110 | Kelp gull | Enteritidis | 2012 |
| SAL4655 | CFSAN024753 | LIOV00000000 | SEN111 | Kelp gull | Enteritidis | 2012 |
| SAL4656 | CFSAN024754 | LIOU00000000 | SEN112 | Kelp gull | Enteritidis | 2012 |
| SAL4657 | CFSAN024755 | LIOT00000000 | SEN127 | Kelp gull | Enteritidis | 2012 |
| SAL4658 | CFSAN024756 | JWQW00000000 | SAG1 | Penguin | Agona | 2012 |
| SAL4659 | CFSAN024757 | JWQV00000000 | SAG2 | Kelp Gull | Agona | 2012 |
| SAL4660 | CFSAN024758 | JWQU00000000 | SAG3 | Penguin | Agona | 2012 |
| SAL4661 | CFSAN024759 | JWQT00000000 | SAG4 | Human | Agona | 2012 |
| SAL4662 | CFSAN024760 | JWQS00000000 | SAG5 | Human | Agona | 2012 |
| SAL4663 | CFSAN024761 | JWQR00000000 | SAG6 | Penguin | Agona | 2012 |
| SAL4664 | CFSAN024762 | [JWQQ01000000](https://www.ncbi.nlm.nih.gov/sites/entrez?db=Nucleotide&cmd=Search&term=JWQQ01000000) | SAN1 | Kelp Gull | Anatum | 2012 |
| SAL4665 | CFSAN024763 | JWQP00000000 | SAN3 | Human | Anatum | 2012 |
| SAL4666 | CFSAN024764 | JWQO00000000 | SAN4 | Human | Anatum | 2012 |
| SAL4667 | CFSAN024765 | JWQN00000000 | SBR1 | Sea Lion | Brandenburg | 2012 |
| SAL4669 | CFSAN024767 | JWQM00000000 | SDU1 | Kelp Gull | Dublin | 2012 |
| SAL4670 | CFSAN024768 | JWQL00000000 | SDU2 | Human | Dublin | 2012 |
| SAL4671 | CFSAN024769 | JWQK00000000 | SDU3 | Human | Dublin | 2012 |
| SAL4672 | CFSAN024770 | JWQJ00000000 | SHA1 | Sea Lion | Havana | 2010 |
| SAL4673 | CFSAN024771 | JWQI00000000 | SHA2 | Garuma Gull | Havana | 2012 |
| SAL4674 | CFSAN024772 | [JWQH00000000](https://www.ncbi.nlm.nih.gov/sites/entrez?db=Nucleotide&cmd=Search&term=NZ_JWQH00000000) | SHE1 | Kelp Gull | Heidelberg | 2012 |
| SAL4675 | CFSAN024773 | JWQG00000000 | SHE2 | Kelp Gull | Heidelberg | 2012 |
| SAL4676 | CFSAN024774 | JWQF00000000 | SHE3 | Kelp Gull | Heidelberg | 2012 |
| SAL4678 | CFSAN024776 | JWQE00000000 | SHE5 | Human | Heidelberg | 2012 |
| SAL4679 | CFSAN024777 | JWQD00000000 | SHE6 | Human | Heidelberg | 2012 |
| SAL4680 | CFSAN024778 | JWQC00000000 | SIN1 | Kelp Gull | Infantis | 2010 |
| SAL4681 | CFSAN024779 | JWQB00000000 | SIN2 | Kelp Gull | Infantis | 2010 |
| SAL4682 | CFSAN024780 | JWQA00000000 | SIN3 | Kelp Gull | Infantis | 2012 |
| SAL4683 | CFSAN024781 | JWPZ00000000 | SIN6 | Human | Infantis | 2012 |
| SAL4684 | CFSAN024715 | JWRH00000000 | SIN7 | Human | Infantis | 2012 |
| SAL4685 | CFSAN024716 | JWRG00000000 | SLI1 | Sea Lion | Livingstone | 2012 |
| SAL4686 | CFSAN024717 | JWRF00000000 | SLI2 | Sea Lion | Livingstone | 2012 |
| SAL4687 | CFSAN024718 | JWRE00000000 | SSE1 | Kelp Gull | Senftenberg | 2010 |
| SAL4688 | CFSAN024719 | JWRD00000000 | SSE2 | Kelp Gull | Senftenberg | 2011 |
| SAL4690 | CFSAN024721 | JWRB00000000 | SSE4 | Human | Senftenberg | 2012 |
| SAL4691 | CFSAN024722 | JWRA00000000 | SSE5 | Human | Senftenberg | 2012 |
| SAL4692 | CFSAN024723 | JWQZ00000000 | SSE6 | Kelp Gull | Senftenberg | 2012 |
| SAL4693 | CFSAN024724 | JWQY00000000 | SSE7 | Kelp Gull | Senftenberg | 2012 |
| SAL4694 | CFSAN024725 | JWQX00000000 | SGB1 | Kelp Gull | Paratyphi B | 2012 |

All strains were isolated in Chile

**Supplementary Table 2. *Salmonella* isolates used in the screening of the detected plasmid (n=113)**

| **Isolate** | **Serotype^1^** | **Origin^2^** | **Plasmid ^3^** |
| --- | --- | --- | --- |
| DR-001 | Enteritidis | Poultry | - |
| DR-002 | Enteritidis | Poultry | - |
| DR-003 | Enteritidis | Poultry | - |
| DR-004 | Tennessee | Poultry | - |
| DR-005 | Infantis | Poultry | - |
| DR-006 | Infantis | Poultry | - |
| DR-007 | Typhimurium | Poultry | - |
| DR-008 | Enteritidis | Poultry | - |
| DR-009 | Typhimurium | Poultry | - |
| DR-012 | Kentucky | Poultry | - |
| DR-013 | Not identified | Poultry | - |
| DR-014 | Mbandaka | Poultry | - |
| DR-015 | Falkensee | Poultry | - |
| DR-016 | Enteritidis | Poultry | - |
| DR-017 | Enteritidis | Poultry | - |
| DR-018 | Enteritidis | Poultry | - |
| DR-019 | Enteritidis | Poultry | - |
| DR-020 | Enteritidis | Poultry | - |
| DR-021^4^ | Hadar | Poultry | Present^4^ |
| DR-022^4^ | Hadar | Poultry farm environment | Present^4^ |
| DR-023 | Typhimurium | Poultry | - |
| DR-024 | Typhimurium | Poultry | - |
| DR-025 | Typhimurium | Poultry | - |
| DR-026 | Typhimurium | Poultry | - |
| DR-027 | Typhimurium | Poultry | - |
| DR-028 | Enteritidis | Poultry | - |
| DR-029 | Infantis | Poultry | - |
| DR-030 | Infantis | Poultry | - |
| DR-031 | Infantis | Poultry | - |
| DP-006 | Not identified | reptile | - |
| DP-007 | Not identified | reptile | - |
| DP-009 | Not identified | reptile | - |
| RT 023 | B | wild bird | - |
| RT 024 | B | wild bird | Present |
| DP-010 | Not identified | reptile | - |
| DP-011 | Not identified | reptile | - |
| DP-013 | Not identified | reptile | - |
| DP-014 | Not identified | reptile | - |
| DP-015 | Not identified | reptile | - |
| DP-016 | Not identified | reptile | - |
| DP-017 | Not identified | reptile | - |
| PS 001 | Typhimurium | horse | - |
| PS 002 | Typhimurium | horse | - |
| PS 003 | Typhimurium | horse | - |
| PS 004 | Typhimurium | horse | - |
| PS 005 | Typhimurium | horse | - |
| PS 006 | Typhimurium | horse | - |
| PS 007 | Typhimurium | horse | - |
| PS 008 | Typhimurium | horse | - |
| PS 009 | Typhimurium | horse | - |
| PS 010 | Typhimurium | horse | - |
| PS 011 | Infantis | horse | - |
| PS 012 | Typhimurium | horse | - |
| PS 014 | Typhimurium | horse | - |
| PS 015 | Typhimurium | horse | - |
| PS 016 | Typhimurium | horse | - |
| PS 017 | Typhimurium | horse | - |
| PS 018 | Typhimurium | horse | - |
| PS 019 | Typhimurium | horse | - |
| PS 020 | Typhimurium | horse | - |
| PS 021 | Infantis | horse | - |
| PS 022 | Infantis | horse | - |
| PS 023 | Typhimurium | horse | - |
| PS 024 | Typhimurium | horse | - |
| PS 025 | Typhimurium | horse | - |
| PS 013 | Typhimurium | horse | Present |
| DP-001 | Not identified | reptile | - |
| DP-002 | Not identified | reptile | - |
| DP-004 | Not identified | reptile | - |
| DP-005 | Not identified | reptile | - |
| DP-008 | Not identified | reptile | - |
| DP-012 | Not identified | reptile | - |
| DP-003 | Not identified | reptile | - |
| DR 041 | Not identified | pig | - |
| DR 043 | Not identified | pig | - |
| DR 044 | Not identified | pig | - |
| DR 045 | Not identified | pig | - |
| DR 046 | Not identified | pig | - |
| DR 047 | Not identified | pig | - |
| DR 039^4^ | Typhimurium | pig | Present^4^ |
| DR 040^4^ | Typhimurium | pig | Present^4^ |
| DR 042 | B | pig | Present |
| RT 022 | B | wild bird | Present |
| RT 002 | Not identified | wild bird | - |
| RT 019 | Not identified | wild bird | - |
| FD 016 | Typhimurium | Cattle | - |
| FD 053 | Not identified | Cattle | - |
| FD P7-6 | Heidelberg | Cattle | - |
| RT 001 | Not identified | Thylamys elegans (marsupial) | - |
| RT 003 | Not identified | Thylamys elegans (marsupial) | - |
| RT 004 | Not identified | Thylamys elegans (marsupial) | - |
| RT 006 | Not identified | Thylamys elegans (marsupial) | - |
| RT 007 | Not identified | Thylamys elegans (marsupial) | - |
| RT 011 | Not identified | Thylamys elegans (marsupial) | - |
| RT 014 | Not identified | Thylamys elegans (marsupial) | - |
| RT 015 | Not identified | Thylamys elegans (marsupial) | - |
| RT 016 | Not identified | Thylamys elegans (marsupial) | - |
| RT 017 | Not identified | Thylamys elegans (marsupial) | - |
| RT 018 | Not identified | Thylamys elegans (marsupial) | - |
| RT 020 | Not identified | Thylamys elegans (marsupial) | - |
| RT 008 | C1 | wild bird | - |
| RT 009 | C1 | wild bird | - |
| RT 010 | C1 | wild bird | - |
| RT 012 | C1 | wild bird | - |
| RT 013 | C1 | wild bird | - |
| DR 038 | Enteritidis | wild bird | - |
| DR-032 | Heidelberg | wild bird | - |
| DR-033 | Infantis | wild bird | - |
| DR-034 | Heidelberg | wild bird | - |
| DR-035 | Heidelberg | wild bird | - |
| DR-036 | Heidelberg | wild bird | - |
| DR-037 | Heidelberg | wild bird | - |
| RT 025 | Not identified | wild bird | - |

^1^ Serotype was predicted using a molecular serotyping scheme described at Ranieri *et al.* 2013. In isolates with only serogroup or not identified, the serotype was not possible to be determined with the scheme.

^2^ All samples were obtained in Chile. Samples from horses were also obtained from the environment of horse hospitals

^3^ Two PCR reactions were used to determine the presence of the plasmid

^4^Isolates were sequenced to obtain plasmid sequences. New identified plasmids were named after the isolate name.

**Supplementary Table 3.** Previously reported plasmids used for genetic comparison.

| Plasmid | Size (bp) | Acc. Number | Bacterial host | Isolation country | Isolation year | Reference |
| --- | --- | --- | --- | --- | --- | --- |
| pMK101 | 2,750 | HM070380.1 | *Salmonella* 6,7:d:- | Colombia | 2007 | (Karczmarczyk *et al.* 2010) |
| pSGI15 | 2,699 | FN428572.1 | *Salmonella* Typhimurium | Netherlands | 2001-2006 | (Hammerl *et al.*2010) |
| pPAB19-4 | 2,702 | JN995611.1 | *Salmonella* sp | Argentina | 2006 | (Tran *et al.*2012) |
| pN44358F | 2,699 | KY991368.1 | *Salmonella* Muenchen | United States | 2013 | (Tyson *et al.*2017) |
| pECY6-7 | 2,699 | GQ374156.1 | *E. coli* | Peru | 2005 | (Pallecchi *et al.*2010) |
| pHAD28 | 2,617 | KU674895.1 | *Salmonella* Hadar | Germany | 2010 | (Fiegen *et al.*2017) |

**Supplementary Table 4**. Distance Matrix inferred by a ClustalW alignment of the plasmids nucleotide sequences.

|  | **pDR021** | **pDR039** | **pDR040** | **pSAL4679** | **pDR022** | **pSAL4674** | **pSAL4678** | **pSAL4688** | **pSAL4630** | **pSAL4629** | **pPAB19-4** | **pHAD28** | **pECY6-7** | **pN44358F** | **pSGI15** | **pMK101** |
| --- | --- | --- | --- | --- | --- | --- | --- | --- | --- | --- | --- | --- | --- | --- | --- | --- |
| **pDR021** |  | 98.2 | 98.2 | 97.2 | 99.9 | 97.2 | 97.2 | 97.2 | 97.2 | 97.2 | 97.2 | 93.7 | 95.5 | 95.5 | 95.5 | 94.4 |
| **pDR039** | 98.2 |  | 100.0 | 95.5 | 98.2 | 95.5 | 95.5 | 95.5 | 95.5 | 95.5 | 95.5 | 92.0 | 93.9 | 93.9 | 93.9 | 92.8 |
| **pDR040** | 98.2 | 100.0 |  | 95.5 | 98.2 | 95.5 | 95.5 | 95.5 | 95.5 | 95.5 | 95.5 | 92.0 | 93.9 | 93.9 | 93.9 | 92.8 |
| **pSAL4679** | 97.2 | 95.5 | 95.5 |  | 97.2 | 98.4 | 98.4 | 98.4 | 98.4 | 98.4 | 98.4 | 94.8 | 96.7 | 96.7 | 96.7 | 95.5 |
| **pDR022** | 99.9 | 98.2 | 98.2 | 97.2 |  | 97.2 | 97.2 | 97.2 | 97.2 | 97.2 | 97.2 | 93.7 | 95.5 | 95.5 | 95.5 | 94.4 |
| **pSAL4674** | 97.2 | 95.5 | 95.5 | 98.4 | 97.2 |  | 100.0 | 100.0 | 100.0 | 100.0 | 100.0 | 96.4 | 98.3 | 98.3 | 98.3 | 97.1 |
| **pSAL4678** | 97.2 | 95.5 | 95.5 | 98.4 | 97.2 | 100.0 |  | 100.0 | 100.0 | 100.0 | 100.0 | 96.4 | 98.3 | 98.3 | 98.3 | 97.1 |
| **pSAL4688** | 97.2 | 95.5 | 95.5 | 98.4 | 97.2 | 100.0 | 100.0 |  | 100.0 | 100.0 | 100.0 | 96.4 | 98.3 | 98.3 | 98.3 | 97.1 |
| **pSAL4630** | 97.2 | 95.5 | 95.5 | 98.4 | 97.2 | 100.0 | 100.0 | 100.0 |  | 100.0 | 100.0 | 96.4 | 98.3 | 98.3 | 98.3 | 97.1 |
| **pSAL4629** | 97.2 | 95.5 | 95.5 | 98.4 | 97.2 | 100.0 | 100.0 | 100.0 | 100.0 |  | 100.0 | 96.4 | 98.3 | 98.3 | 98.3 | 97.1 |
| **pPAB19-4** | 97.2 | 95.5 | 95.5 | 98.4 | 97.2 | 100.0 | 100.0 | 100.0 | 100.0 | 100.0 |  | 96.4 | 98.3 | 98.3 | 98.3 | 97.1 |
| **pHAD28** | 93.7 | 92.0 | 92.0 | 94.8 | 93.7 | 96.4 | 96.4 | 96.4 | 96.4 | 96.4 | 96.4 |  | 94.7 | 94.7 | 94.7 | 93.5 |
| **pECY6-7** | 95.5 | 93.9 | 93.9 | 96.7 | 95.5 | 98.3 | 98.3 | 98.3 | 98.3 | 98.3 | 98.3 | 94.7 |  | 100.0 | 100.0 | 98.0 |
| **pN44358F** | 95.5 | 93.9 | 93.9 | 96.7 | 95.5 | 98.3 | 98.3 | 98.3 | 98.3 | 98.3 | 98.3 | 94.7 | 100.0 |  | 100.0 | 98.0 |
| **pSGI15** | 95.5 | 93.9 | 93.9 | 96.7 | 95.5 | 98.3 | 98.3 | 98.3 | 98.3 | 98.3 | 98.3 | 94.7 | 100.0 | 100.0 |  | 98.0 |
| **pMK101** | 94.4 | 92.8 | 92.8 | 95.5 | 94.4 | 97.1 | 97.1 | 97.1 | 97.1 | 97.1 | 97.1 | 93.5 | 98.0 | 98.0 | 98.0 |  |

Estimation were made with plasmid sequences found in this study and previously reported sequences. The calculations were run with ClustalW in Geneious Prime 2019.1.1 (Biomatter, New Zealand).

**Supplementary Table 5:** Plasmid replicons present in *Salmonella* genomes in the study.

| Strain | Replicon | Identity | Query / Template length | Contig | Position in contig | Accession number |
| --- | --- | --- | --- | --- | --- | --- |
| SAL4629 | IncFIB(S) | 98.91 | 643 / 643 | 20 | 34322..34964 | [FN432031](http://www.ncbi.nlm.nih.gov/nuccore/FN432031) |
|  | IncFII(S) | 100 | 262 / 262 | 21 | 9755..10016 | [CP000858](http://www.ncbi.nlm.nih.gov/nuccore/CP000858) |
| SAL4630 | IncFIB(S) | 98.91 | 643 / 643 | 34 | 7047..7689 | [FN432031](http://www.ncbi.nlm.nih.gov/nuccore/FN432031) |
|  | IncFII(S) | 100 | 262 / 262 | 31 | 3566..3827 | [CP000858](http://www.ncbi.nlm.nih.gov/nuccore/CP000858) |
|  | IncN | 99.81 | 514 / 514 | 22 | 247..760 | [AY046276](http://www.ncbi.nlm.nih.gov/nuccore/AY046276) |
| SAL4675 | IncX1 | 98.93 | 374 / 374 | 18 | 31865..32238 | [EU370913](http://www.ncbi.nlm.nih.gov/nuccore/EU370913) |
| SAL4676 | IncI1 | 99.3 | 142 / 142 | 12 | 67302..67443 | [AP005147](http://www.ncbi.nlm.nih.gov/nuccore/AP005147) |
|  | IncX1 | 98.93 | 374 / 374 | 14 | 25139..25512 | [EU370913](http://www.ncbi.nlm.nih.gov/nuccore/EU370913) |
| SAL4678 | None | | | | | |
| SAL4679 | None | | | | | |
| SAL4674 | None |  |  |  |  |  |
| SAL4688 | IncL/M(pOXA-48) | 95.2 | 666 / 661 | 30 | 16154..16817 | [JN626286](http://www.ncbi.nlm.nih.gov/nuccore/JN626286) |
| DR021 | Col440I | 95.18 | 83 / 114 | 67 | 1..83 | [CP023920.1](http://www.ncbi.nlm.nih.gov/nuccore/CP023920.1) |
| DR022 | Col440I | 95.18 | 83 / 114 | 68 | 1..83 | [CP023920.1](http://www.ncbi.nlm.nih.gov/nuccore/CP023920.1) |
| DR039 | None |  |  |  |  |  |
| DR040 | None |  |  |  |  |  |

Search was performed with PlasmidFinder, DTU with default parameters for Enterobacteriaceae (Carattoli et al, 2014)

**Supplementary Figure 1**. Phylogeny inferred from 6 Chilean plasmids sequences and 6 similar plasmids from the NBCI database.


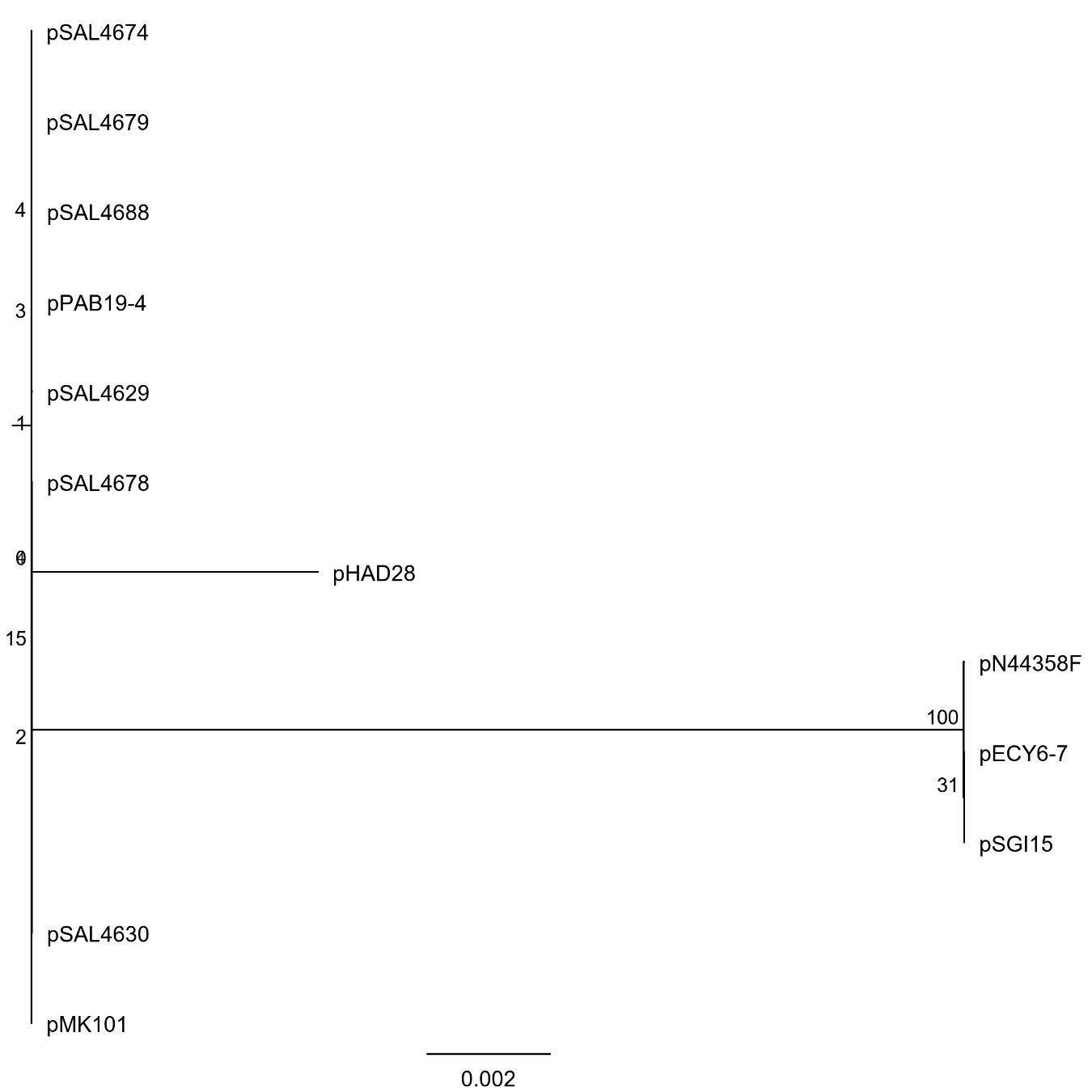


Alignment was performed with ClustalW with default parameters, and phylogeny was inferred with RAxML (Stamatakis, Hoover and Rougemont 2008) in Geneious (Biomatters, New Zealand) with 1,000 replicates. Phylogeny was used to define sequences to be used for primer design.

**
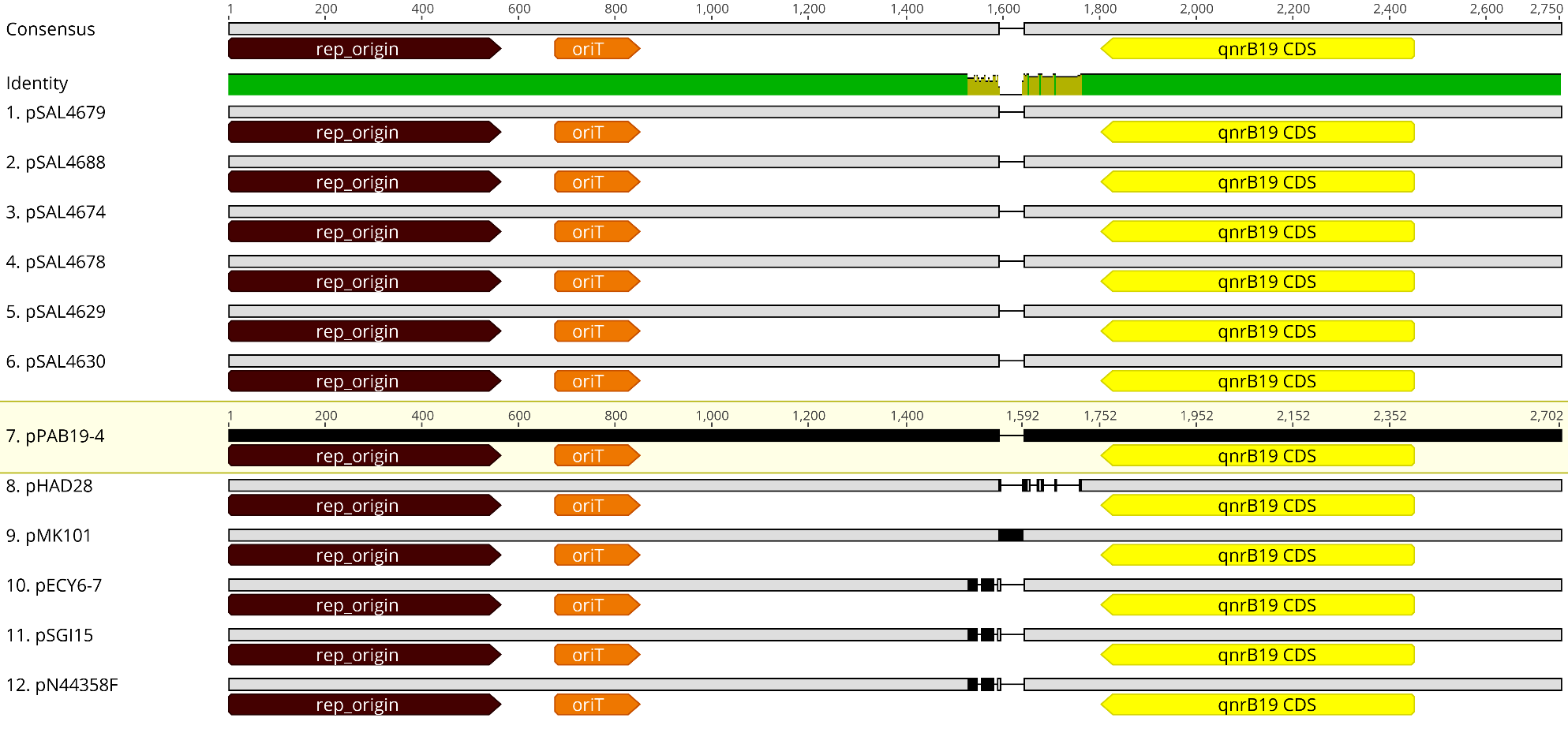
**

**Supplementary Figure 2:** Nucleotide alignment of *qnr*B19-carrying plasmids isolated in Chile (n=6) and from NCBI.

Alignment was performed with ClustalW using default parameters in Geneious Prime. Non-identities are located between nucleotides 1,529 and 1761 using consensus sequence as reference.

Green bar at the “consensus identity sequence” represents 100% identity, and red indicates <30% identity.

**
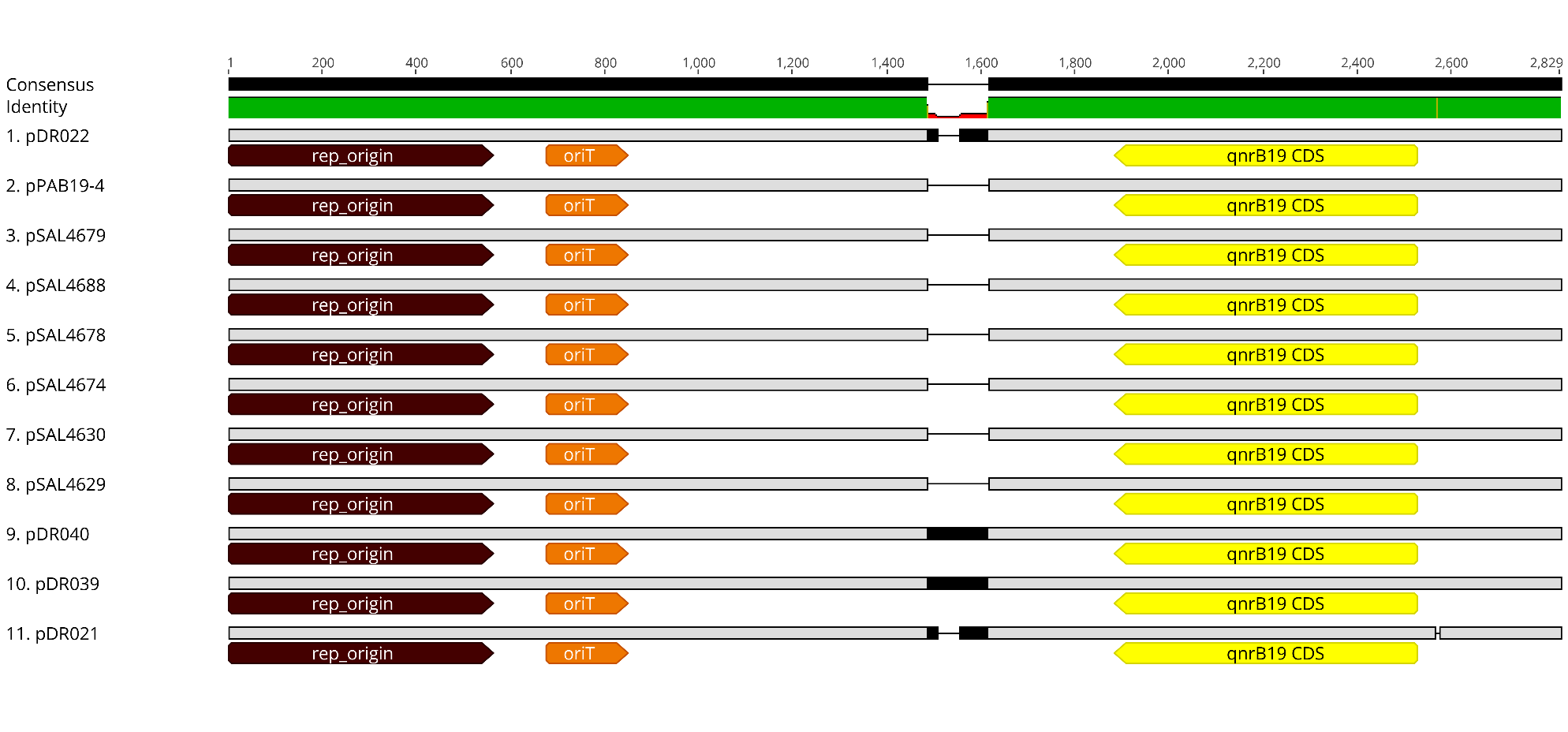
Supplementary Figure 3:** Nucleotide alignment of *qnr*B19-carrying plasmids isolated in Chile (n=10) and pPAB-19.

Alignment was performed with ClustalW using default parameters in Geneious Prime. Insertions are located in nucleotide 1,496 (Plasmid pPAB19-4 as a reference).

Green bar at the “consensus identity sequence” represents 100% identity, and red indicates <30% identity.
